# Implementation of a Novel Agitated Behavior Score and Its Association with Code Violet Activation: A Case Series

**DOI:** 10.5811/cpcem.62328

**Published:** 2026-08-03

**Authors:** Negin Ceraolo, Julianna Sandine, Bethany Crouse, Jessica Krizo, Erin Simon

**Affiliations:** * Cleveland Clinic Akron General, Department of Emergency Medicine, Akron, Ohio; † Northeast Ohio Medical University, Rootstown, Ohio; ‡ Cleveland Clinic Akron General, Department of Pharmacy, Akron, Ohio; § Cleveland Clinic Akron General, Department of Health Sciences, Akron, Ohio

**Keywords:** agitation, assessment tool, pharmacological sedation, physical restraint, case report

## Abstract

**Introduction:**

Agitation in the emergency department (ED) poses a significant safety concern for staff and patients. Multidisciplinary responses, such as Code Violet activations, are common but resource-intensive. Early identification and intervention may reduce escalation, but ED-specific data are limited. The objective of the study was to evaluate the implementation of a novel agitated behavior score (ABS) and assess whether higher ABS scores are associated with increased risk of Code Violet activation.

**Case Series:**

This prospective observational study included adult patients admitted to a behavioral health unit in a community teaching hospital ED. Trained staff administered a novel 25-point ABS incorporating altered mentation, verbal agitation, and motor agitation. The primary outcome was Code Violet activation; secondary outcomes included pharmacologic interventions and substance use. Among 83 patients, 27 (33%) experienced ≥ 1 Code Violet activation. And those patients with Code Violet activation had higher initial ABS scores (mean 10.9 [6.35] versus 4.6 [4.43]; *P* <.001). Overall rates of psychotropic medication use were similar between groups; however, time to first medication was longer in the Code Violet group (4.0 versus 2.7 hours). Patients with Code Violet activations more frequently received parenteral medications, whereas those without the code activation more commonly received oral agents.

**Conclusion:**

The agitated behavior score was associated with Code Violet activation and may help identify patients at greater risk of behavioral escalation. While it measures current agitation rather than predicting future agitation, its structured format may facilitate early recognition and management of agitation.

## INTRODUCTION

Agitation is a common and potentially dangerous behavioral presentation in the emergency department (ED), affecting approximately 2–3% of visits.^1^,^2^,^9^ When unrecognized or inadequately managed, agitation can escalate to violence, delay medical care, and increase reliance on sedative medications or physical restraints, interventions that pose significant safety risks to both patients and staff.^6^–^8^ Initial management should prioritize nonpharmacologic strategies, particularly verbal de-escalation.^3^–^5^ When these measures fail, ED teams may activate a Code Violet, a multidisciplinary emergency response for violent or combative patients. Such activations frequently necessitate pharmacologic or physical restraint, leading to increased patient morbidity and resource use.^11^–^15^

Despite the critical importance of early agitation recognition, few tools exist to assess agitation, specifically in the ED setting. Commonly used scales, including the Richmond Agitation and Sedation Scale (RASS), Agitated Behavior Scale (ABS), Positive and Negative Syndrome Scale, Excited Component (PANSS-EC), and Behavioral Activity Rating Scale (BARS), focus on current agitation severity, and were developed for psychiatric or intensive care environments.^10^

In this pilot study we evaluated a novel agitated behavior score (ABS) designed for use in the ED behavioral health unit. We compared ABS scores among patients who experienced Code Violet activations with those who did not, aiming to determine whether the ABS could aid in early risk stratification and escalation prevention. This prospective observational study was conducted in the ED of an urban, Level I trauma center, community teaching hospital. The study focused on patients admitted to the ED’s behavioral health unit, which specializes in the evaluation and management of psychiatric and behavioral presentations. Eligible patients were adults (≥ 18 years of age) triaged to the ED behavioral health unit between May–November 2024. A convenience sample of patients in this unit were screened and scored using the ABS upon arrival. No exclusion criteria were applied during this pilot phase.

*CPC-EM Capsule*

What do we already know about this clinical entity?

*Agitation in the emergency department (ED) is common and dangerous; existing scales assess severity but are not designed for ED-specific risk stratification.*

What makes this presentation of disease reportable?

*This study introduces a novel ED-specific agitated behavior score and demonstrates its association with Code Violet activation risk.*

What is the major learning point?

*Higher agitation scores correlate with escalation risk, and delayed pharmacologic intervention may contribute to Code Violet activation.*

How might this improve emergency medicine practice?

*Structured agitation scoring may enable earlier recognition, timely intervention, and reduce escalation, restraints, and resource-intensive responses.*

### Agitated Behavior Score (ABS)

The novel ABS is a 25-point scale composed of three domains (Image):

- Altered mentation (6 points): impulsivity, attention, hallucinations, mood lability
- Verbal agitation (7 points): verbal threats, loudness, cooperation, outbursts
- Motor agitation (12 points): wandering, physical resistance, pulling at equipment, self-harm behaviors

**Figure 1:**
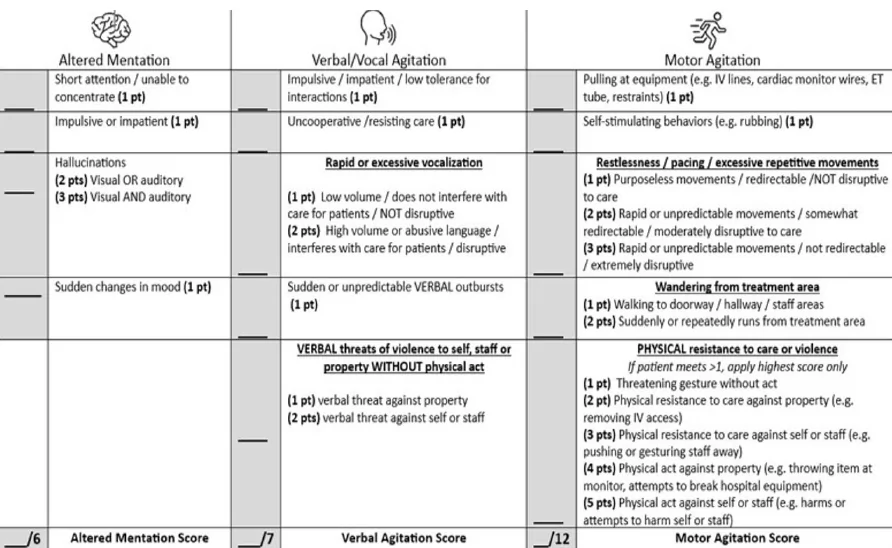
Distribution of psychiatric diagnoses among study participants.

**Figure 2:**
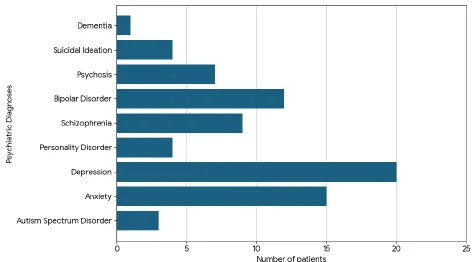
Substance use identified among study participants based on toxicology screening. Frequencies represent the number of patients with positive results for each substance. Patients may have tested positive for multiple substances; therefore, totals exceed 100%.

Each domain was scored through direct observation and interaction by trained clinical staff. In addition, staff assigned a subjective Code Violet risk assessment (low, moderate, high) estimating the likelihood of agitation escalation within the next two hours. Data was abstracted from the electronic health record (EHR) (Epic Systems Corporation, Verona, WI) by a trained data analyst who was blinded to the study hypothesis. Demographic data, psychiatric history (Figure 1), substance use (Figure 2), ED course, medication administration (including time, route, and type), Code Violet activation, and lab results (eg, urine drug screens, serum alcohol levels) were abstracted retrospectively from the EHR.

Psychiatric conditions were identified based on documentation in the EHR. Categories are not mutually exclusive, and some patients had multiple coexisting diagnoses. The primary outcome was Code Violet activation during the ED encounter (binary: yes/no). Secondary outcomes included time to medication administration, documentation of substance use, psychiatric diagnosis, and subjective Code Violet risk assessment. We compared mean ABS scores between patients with and without Code Violet activations using a two-tailed *t*-test. Descriptive statistics were used for demographic and clinical variables. A *P*-value <. .05 was considered statistically significant.

## CASE SERIES

The study included 83 patients, 18–87 years of age. The cohort included 57 males (69%) and 26 females (31%); 45 patients (54.2%) had positive toxicology screens; 12 (14.5%) presented with acute alcohol intoxication. A total of 27 patients (33%) experienced at least one Code Violet activation, and among these 11 patients had multiple activations. Patients who had Code Violet activations showed a higher mean ABS score of 10.9 (6.35), compared to a mean ABS score of 4.6 (4.43) in patients without Code Violet activations. This difference between the two groups was statistically significant (*t* = 6.75; *P* = 1.73 × 10^−9^). A total of 172 ABS assessments were completed. Patients with higher ABS were more likely to be subjectively labeled “high risk” for Code Violet. Among non-Code Violet patients, five were still assessed as “high risk.”

**Figure 3:**
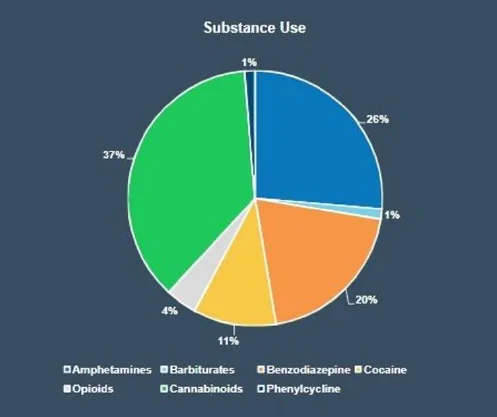
Time to first pharmacologic intervention in patients with and without Code Violet activation. Patients who experienced Code Violet activation had a longer mean time to initial medication administration compared to those without Code Violet activation (4.0 versus 2.7 hours). *avg*, average; *CV*, Code Violet; *h*, hours.

A total of 47 patients (56.6%) received psychotropic medications, with no significant difference in administration rates between patients with and without Code Violet activations. The mean time to first medication was longer in the Code Violet group, at 4.0 hours compared to 2.7 hours (see Figure 3). In the Code Violet group, commonly administered medications included parenteral droperidol (56%), haloperidol (17%), midazolam (19%), and lorazepam (11%). In contrast, the non-Code Violet group more frequently received oral medications, particularly olanzapine (30%) and lorazepam. Of the 13 patients who were initially given oral medications, only three (23.1%) went on to experience a Code Violet activation.

A total of 45 patients (54.2%) tested positive for drugs, including 16 who experienced Code Violet activations. Twelve patients presented with alcohol intoxication, of whom six (50%) had Code Violet activations that occurred on average within 38 minutes of arrival to the ED. The range of detected illicit substances included amphetamines in 20 patients (44.4%), barbiturates in one patient (2.2%), benzodiazepines in 15 patients (33.3%), cocaine in eight patients (17.8%), opiates in three patients (6.7%), cannabinoids in 28 patients (62.2%), and phencyclidine in one patient (2.2%) (see Figure 2).

## DISCUSSION

In this pilot study, higher ABS values were significantly associated with Code Violet activations, indicating that patients with greater behavioral dysregulation at triage were more likely to require multidisciplinary intervention. Subjective Code Violet risk ratings closely aligned with actual Code Violet occurrences, supporting internal consistency and construct validity. Earlier administration of oral medications appeared to mitigate escalation, whereas patients requiring parenteral agents were more likely to experience agitation severe enough to trigger a Code Violet (Figure 3).

An important finding in this study was the longer time to pharmacologic intervention in patients who ultimately required Code Violet activation. This delay is notable given that these patients demonstrated higher levels of agitation on initial assessment. Several factors may explain this pattern. Diagnostic uncertainty, particularly in patients without clear intoxication or psychiatric history, may have contributed to hesitation in early medication administration. Additionally, initial agitation in the Code Violet group may have been intermittently manageable with verbal de-escalation, delaying escalation to pharmacologic treatment. Operational barriers, including staff availability, safety considerations, and time required to mobilize resources, may have further contributed to delays. Variability in clinician comfort with early sedation in undifferentiated agitation may also play a role. These findings highlight an opportunity for earlier, standardized intervention strategies in high-risk patients identified by elevated ABS scores.

These findings reinforce the importance of structured assessment in managing agitation, a behavioral presentation affecting approximately 2–3% of ED visits.^1^,^2^ Unrecognized or inadequately managed agitation can escalate to violence, delay care, and increase reliance on physical restraint or sedative medications, each posing risks to both patients and staff.^6^–^8^ Several existing agitation scales, such as the RASS, ABS, PANSS-EC, and BARS, were developed for psychiatric or intensive care settings and primarily measure current agitation severity rather than risk of escalation.^10^ The novel ABS was designed specifically for use in the ED behavioral health environment, emphasizing rapid administration and broad usability among nursing and ancillary staff.

While the agitated behavior score quantifies present behavioral intensity rather than predicting future agitation in a time-dependent manner, its structured approach facilitates consistent assessment, enhances communication between physicians, and promotes early recognition of risk. Early identification allows for proactive, less invasive interventions such as verbal de-escalation or timely oral pharmacologic treatment.^3^,^4^ Incorporating a standardized agitation assessment tool into the ED workflow may improve patient and staff safety. Objective risk scoring can guide targeted allocation of resources such as security support or medication preparation and may reduce reliance on emergent parenteral administration or physical restraint. Structured documentation of agitation levels also supports departmental quality improvement initiatives and provides measurable benchmarks for future intervention studies.

## LIMITATIONS

This study has several limitations. The small sample size and single-center convenience sampling design may limit generalizability. Subjective elements within the Code Violet risk assessment could have introduced observer bias. Additionally, this pilot did not include comparison with established agitation scales (eg, RASS, BARS) or clinician gestalt, limiting external validation. Data abstraction from the EHR may also have been subject to documentation variability.

Future studies should validate the ABS in larger, multicenter cohorts and assess interrater reliability across different clinician roles. Comparative analyses with existing agitation scales and clinician assessments would provide additional validity data. Longitudinal studies using serial ABS measurements could help determine whether the tool predicts agitation escalation in real time. Furthermore, protocolized interventions triggered by elevated ABS scores such as early oral pharmacologic treatment or increased monitoring should be evaluated for their effect on safety outcomes and resource use.

## CONCLUSION

The agitated behavior score was strongly associated with Code Violet activation in an ED behavioral health population. Its structured, rapid format may facilitate early identification of patients at higher risk of behavioral escalation, enabling timely and less invasive management strategies. Although the ABS measures current rather than predictive agitation, it shows promise as a standardized, scalable approach to agitation risk stratification and escalation prevention in emergency care settings.

## Figures and Tables

**Image.**
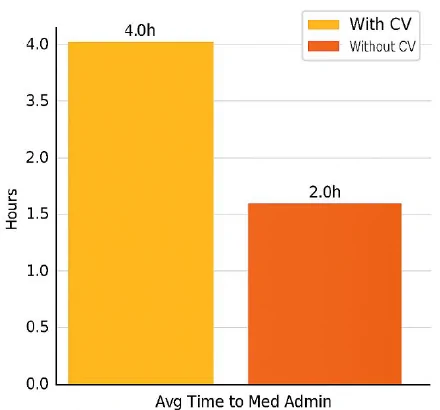
Agitated behavior score rubric designed for use in an emergency department behavioral health unit. *ET*, endotracheal tube; *IV*, intravenous; *pt(s)*, point(s).

## References

[1] Sharma NP, Huecker MR (2025). Agitation. StatPearls [Internet].

[2] Miner JR, Klein LR, Cole JB (2018). The characteristics and prevalence of agitation in an urban county emergency department. Ann Emerg Med.

[3] Richmond JS, Berlin JS, Fishkind AB (2012). Verbal de-escalation of the agitated patient: consensus statement of the American Association for Emergency Psychiatry Project Beta De-Escalation Workgroup. West J Emerg Med.

[4] Holloman GH, Zeller SL (2012). Overview of Project BETA: best practices in evaluation and treatment of agitation. West J Emerg Med.

[5] Roppolo LP, Morris DW, Khan F (2020). Improving the management of acutely agitated patients in the emergency department through implementation of Project BETA (Best Practices in the Evaluation and Treatment of Agitation). J Am Coll Emerg Physicians Open.

[6] Cohen S, Meyer A, Ifrach N (2024). Physical restraint and associated agitation. Nurs Crit Care.

[7] Knox DK, Holloman GH (2012). Use and avoidance of seclusion and restraint: consensus statement of the American Association for Emergency Psychiatry Project Beta Seclusion and Restraint Workgroup. West J Emerg Med.

[8] Yap CYL, Taylor DM, Kong DCM (2019). Risk factors for sedation-related events during acute agitation management in the emergency department. Acad Emerg Med.

[9] American College of Emergency Physicians (2022). Emergency department violence poll results.

[10] Thiessen MEW, Godwin SA, Hatten BW (2024). Clinical policy: critical issues in the evaluation and management of adult out-of-hospital or emergency department patients presenting with severe agitation. Ann Emerg Med.

[11] Miner JR, Klein LR, Cole JB (2018). The characteristics and prevalence of agitation in an urban county emergency department. Ann Emerg Med.

[12] Gault TI, Gray SM, Vilke GM (2012). Are oral medications effective in the management of acute agitation?. J Emerg Med.

[13] Mullinax S, Shokraneh F, Wilson MP (2017). Oral medication for agitation of psychiatric origin: a scoping review of randomized controlled trials. J Emerg Med.

[14] Knott JC, Taylor DM, Castle DJ (2006). Randomized clinical trial comparing intravenous midazolam and droperidol for sedation of the acutely agitated patient in the emergency department. Ann Emerg Med.

[15] Kynoch K, Wu CJ, Chang AM (2011). Interventions for preventing and managing aggressive patients admitted to an acute hospital setting: a systematic review. Worldviews Evid Based Nurs.

